# Pedunculoside protects against LPS-induced mastitis in mice by inhibiting inflammation and maintaining the integrity of blood-milk barrier

**DOI:** 10.18632/aging.203357

**Published:** 2021-08-12

**Authors:** Xingchi Kan, Guiqiu Hu, Bingxu Huang, Wenjin Guo, Yaping Huang, Yingsheng Chen, Ping Xu, Xiangyu Cai, Shoupeng Fu, Juxiong Liu

**Affiliations:** 1 Department of Theoretic Veterinary Medicine, College of Veterinary Medicine, Jilin University, Changchun, Jilin, China

**Keywords:** mastitis, LPS, AKT/NF-κB, MAPK, blood-milk barrier

## Abstract

Mastitis is a disease that seriously threatens the health of the mammary gland after delivery. Pedunculoside (PE) is the main bioactive component of Aquifoliaceae. The purpose of this experiment is to explore the effects of PE on mastitis and its underlying mechanisms. Our research results showed that PE could significantly inhibit the increase in the levels of inflammatory mediators such as TNF-α, IL-6, IL-1β, MPO and iNOS during mastitis. Mechanism studies have found that PE could significantly inhibit the phosphorylation of AKT protein and binds to the ASP-184 site. Further research found that PE also inhibited the activation of AKT's downstream pro-inflammatory signals NF-κB and MAPK. In addition, PE effectively promote the expression of tight junction proteins occludin and claudin-3 during inflammation, maintaining the integrity of the blood-milk barrier. In summary, our research shows that PE inhibits the phosphorylation of AKT/NF-κB and MAPK signals; It also relieves mastitis by repairing the blood-milk barrier.

## INTRODUCTION

Mastitis is one of the three most serious diseases in dairy cows, which causes huge economic losses every year [1]. Nipple muscle relaxation, milk deposition, and environmental pathogenic microbial infections are the main causes of disease [1, 2]. Mastitis can be divided into clinical type and recessive mastitis. Mastitis caused by gram-negative bacteria is a common clinical situation [3]. At present, the clinical effect of preventing and treating mastitis is still not significant, so it is very urgent to find a new food supplement with significant effect, safe and no side effects.

LPS is the main pathogenic factor on the cell wall surface of gram-negative bacteria. It activates inflammatory signals by binding to the TLR4 receptor on the cell surface to promote the production of inflammatory mediators such as IL-6, IL-1β, TNF-α, iNOS, MPO, etc. Amplify the inflammatory signal and aggravate the inflammatory response [4–6]. In addition, LPS will continue to transmit inflammatory signals downward, such as activation of the AKT/NF-κB signaling pathway and MAPK signaling pathway, which will continue to intensify and maintain the inflammatory environment [6, 7]. In addition, the clinical mastitis model constructed by LPS is used as the selection of drug candidates [8, 9], so the LPS-induced mice mastitis model is scientifically reasonable.

Blood-milk barrier is an important biological barrier of mammary gland, which has important biological functions, such as resisting the invasion of pathogens, preventing the loss of milk nutrients, and maintaining the steady-state regulation of mammary tissue microenvironment [9, 10]. Occludin, claudin-3, ZO-1 and other tight junction proteins are the main components of blood-milk barrier. The destruction of blood-milk barrier will increase the risk of mammary disease [3, 11]. Studies have shown that LPS can lead to the destruction of blood-milk barrier during mastitis, such as the decrease of occludin protein expression [9]. Our previous research results show that repairing the blood-milk barrier during the inflammatory response is beneficial to the relief of mastitis, such as promoting the expression of claudin-3 protein, which is beneficial to the relief of mastitis [8]. Therefore, promoting the expression of tight junction protein in the process of mastitis may be an important means to repair the blood-milk barrier and a potential treatment to alleviate mastitis.

Aquifoliaceae has been used as herbal tea and traditional Chinese medicine. Pedunculoside (PE) is the main bioactive component of Aquifoliaceae, a kind of flavonoids, with has a wide range of biological activities [12]. There is increasing evidence that flavonoids are beneficial to health [13]. Studies have shown that PE has a wide range of physiological activities, such as relieving synovial joint inflammation [14], preventing liver damage [15], improving intestinal flora [16], and reducing hyperlipidemia caused by high-fat diet [17] and other functions. However, whether PE has a preventive effect on mastitis has not yet been reported. Therefore, the purpose of this experiment is to explore the effects of PE on mastitis and its underlying mechanisms from the perspectives of inflammation and blood-milk barrier.

## MATERIALS AND METHODS

### Animal

Forty-five 7-week-old mice were purchased from Changsheng Liaoning including 15 male mice and 30 female mice (Liaoning, China). Each cage was divided into 2 females and 1 male mice. After the pregnant female mice were fertilized, the male mice were removed. Mice eat and drink freely, and cycle light and dark for 12 h. The experiment was divided into 5 groups with 6 female mice in each group, namely no treatment group (NT), pedunculoside alone group (PE), model group (Lipopolysaccharide, LPS), pedunculoside treatment group (LPS+PE), and dexamethasone positive control group (LPS+DEX). All animal experiments were performed strictly according to the experimental guidelines [18, 19].

### Construction of mastitis model

The mouse model of mastitis was constructed within 3-10 days after delivery. The mice were injected with 100 μL of sodium pentobarbital (45 mg/kg). The fourth pair of nipples were disinfected with 75% alcohol (Beijing industry group, Beijing, China), and the nipples were lifted with flat forceps and then minus about 1 mm with scissors to expose the milk ducts. Next, a 34 G needle was inserted into the mammary duct, 50 μL of LPS (0.2 mg/mL) (Sigma-Aldrich, Saint Louis, MO, USA) was perfused, and the mammary injected with LPS are gently massaged to make the LPS evenly distributed. Mammary gland samples were collected 24 h after LPS injection for subsequent experiments.

### Management of drug

PE (HPLC ≥ 98%), purchased from Shanghai Yuanye Biology Co., Ltd., dissolved in methanol, stored in -20° C refrigerator. PE (10 mg/kg) was given orally for 7 days [17]. 10 μM PE was used for pre-protection, in the cell experiments. DEX (St. Louis, MO, USA) (5 mg/kg) was administered by intraperitoneal injection.

### H&E

Fresh mammary tissue was fixed in 10% formaldehyde solution (Beijing Industry Group, Beijing, China) for 48 h, and then the tissue block was squared. The next step was alcohol dehydration, transparent xylene, tissue wax impregnation and tissue block embedding. Next, 5 μM thick tissue sections were made.

The prepared paraffin sections are deparaffinized with xylene, and then passed 100%, 100%, 95%, 90%, 80%, 70% alcohol. Then the slices go through hematoxylin and eosin (Beijing Solarbio Science and Technology Co., Ltd.) in sequence, and finally the slices were sealed with neutral gum. According to the degree of mammary interstitial edema of the mammary glands, the integrity of the acinar and the infiltration of inflammatory cells in the acinar, the damage of the mammary tissue was scored. 0: no injury, 1: mild injury, 2: poisoning injury, 3: severe injury, 4: extreme injury.

### Determination of MPO and ELISA

Take fresh mammary tissue into a 2 mL grinding tube and weigh the mammary tissue. 0.1 g of mammary tissue was added with 400 μL of HEPES buffer solution and added with small steel balls for grinding. The grinding condition was 10 min 50 HZ, followed by centrifugation at 12000 rpm/min for 10 min, and the supernatant was taken as the MPO sample. Then add an equal volume of 0.25% CTAC solution to the remaining sediment in a 2 mL EP tube and continue grinding for 10 min, followed by centrifugation at 12000 rpm/min for 10 min to take the supernatant as an ELISA sample. The MPO sample was measured as described previously [8]. ELISA was determined by Solarbio kit (Beijing Solarbio Science and Technology Co., Ltd.), and the experimental method was strictly in accordance with the instructions.

### Cell culture

Mouse mammary epithelial cells (mMECs) was purchased from American Type Culture Collection (ATCC, ATCC® CRL-3063™). DMEM (Gibco, Grand Island, NY 14072, USA) was used for the cultivation of mMECs, and 10% FBS (Clark Bioscience, USA) was added to the medium to ensure the normal growth of cells. mMECs were placed in a cell culture box and cultured under 37° C and 5% CO_2_ conditions. 0.25% trypsin was used for cell digestion. The cell suspension was centrifuged at 1000 rpm/min for 3 min, fresh DMEM medium was added to the cell pellet, and then the cells were evenly seeded in a 60 cm cell culture dish. After the cell fusion density reached 65%, PE was added to the cell culture medium at a concentration of 10 μM. After 1 h, LPS was added to the culture medium at a concentration of 1 μg/mL. The co-stimulation time was 12 h. Finally, cell samples were collected for subsequent experiments.

### CCK8

The mMECs were uniformly seeded in a 96-well plate, and each well contained 5000 cells in 100 μL of DMEM medium (Gibco, NY, USA). After the cells adhere to the wall, different concentrations of PE (0, 1, 10, 500, 1000 μM) are added to stimulate the cells for 12 h. Then added 10 μL of CCK8 solution to each well, and measure the absorbance at 450 nm after 2 h.

### Reverse transcription and quantitative real-time PCR (q-RTPCR)

TRIZOL (Sigma-Aldrich, Saint Louis, MO, USA) was added to the cell sedimentation for lysis for total RNA extraction. The samples were sequentially added with chloroform, isopropanol, 75% DEPC alcohol, and DEPC water. Determine the concentration of total RNA and carry out aliquots. RNA samples are stored in -80° C refrigerator for later use. Oligdt, MLV, RRI, and dNTP mix (Takara, Japan) were added to RNA sequentially. The reverse transcription conditions are: 70° C for 10 min, 42° C for 60 min, and 70° C for 15 min.

Next, perform q-RTPCR, add 10 μL of fluorescent dye SYBR green (Takara, Kyoto, Japan) to cDNA, 1 μL of upstream and downstream primers, and 20 μL of reaction system. The reaction conditions of q-RTPCR are: 95° C for 4 min, 95° C for 15 s, 60° C for 90 s, 40 cycles. The primer sequences used in the reaction are shown in Table 1.

**Table 1:** The primer sequences of *TNF-α, IL-1*β, *IL-6* and β*-actin*.

**Name**	**Primer**	**Length of base pair**
*TNF-α* (Forward)	5’-ACGGCATGGATCTCAAAGAC-3’	116
*TNF-α* (Reverse)	5’-GTGGGTGAGGAGCACGTAGT-3’	
*IL-1β* (Forward)	5’-GCTGCTTCCAAACCTTTGAC-3’	121
*IL-1β* (Reverse)	5’-AGCTTCTCCACAGCCACAAT-3’	
*IL-6* (Forward)	5’-CCGGAGAGGAGACTTCACAG-3’	134
*IL-6* (Reverse)	5’- CAGAATTGCCATTGCACAAC-3’	
*β-actin* (Forward)	5’- ATCACTATTGGCAACGAGCGGTTC-3’	147
*β-actin* (Reverse)	5’-CAGCACTGTGTTGGCATAGAGGTC-3’	

### Western blot

Add protein lysis solution NP40 (Biyuntian, Wuhan, China) to tissue homogenate or cell sedimentation. After centrifugation, the supernatant was taken as a total protein sample, 200 μL of BCA (Biyuntian, Wuhan, China) was added to the protein sample and standard protein, and the protein concentration was determined and SDS was added to aliquot.

Prepare 4% concentrated glue and 12% separating glue. Add ddH_2_O, Acry-Bis, PH 6.8, PH 8.8, 10% SDS, 10% APS, TEMED according to the ratio, as previously described [8]. Next, add the sample to the page-gel at 100 V for 90 min. Tthe PVDF membrane was covered on the page-gel and energized 80 V for 60 min. The PVDF membrane was immersed in 5% milk for 2 h, and then the primary antibody (Cell Signaling Technology, Danvers, MA, USA) was added dropwise on the PVDF membrane in the refrigerator at 4° C overnight [20, 21]. The next day, the primary antibody on the PVDF membrane was washed off, and then the PVDF membrane was immersed in the secondary antibody (goat anti-mice or goat anti-rabbit, Hubei, China) solution for 1 h. Remove the secondary antibody and add the ECL (Applygen Technologies, Beijing, China) solution to obtain protein bands. The first antibody was diluted with 5% BSA at the ratio of 1:1000, p65, p-p65, IκB, P-IκB, β-actin, p-p38, p38, JNK, p-JNK, ERK, p-ERK were purchased from cell signaling technology (Danvers, Ma, USA); iNOS was purchased from Abcam Co., Ltd. (Shanghai, China); Akt, p-Akt were purchased from protentech Co., Ltd. (Wuhan, China). Second antibody (good anti Mie and good anti rabbit) was diluted with 5% milk at the ratio of 1:5000. It were purchased from boster Biological Technology Co., Ltd. (Hubei, China).

### Molecular modelling

The 3D structure of PE was obtained from PubChem https://pubchem.ncbi.nlm.nih.gov/. The initial X-ray crystal structure of AKT was obtained from the Protein Data Bank (PDB) with PDB code: 3ow 3. Demolition with Pymol Divide the small molecule inhibitors that bind to AKT and save them in pbdqt format. Similarly, PE is also saved in pdbqt format in Pymol. Autodock4 calculated the coordinates of the active center of the AKT protein, then set the docking parameters, PE was selected as the ligand, and AKT was selected as the target, and 2.5×10^6^ operations were executed. The binding energy of PE and AKT protein and the amino acid sites of action were derived from analysis of Autodock 4 software. The 3D combination mode picture of AKT and PE was obtained through the software Pymol.

### Determination of the integrity of the blood-milk barrier

Prepare 2 mg/mL FITC (Sigma-Aldrich, Saint Louis, MO, USA) solution with PBS in a 1.5 mL EP tube, then take fresh tissue and immerse it in 500 μL FITC solution, and let it stand for 15 min in the dark, in order to let FITC-albumin free in the matrix between the alveoli shuttle. Then quickly put the 1.5 mL EP tube containing mammary tissue into the liquid ammonia, and then remove the tissue mass in the liquid nitrogen. The mammary tissue block was put into a cryostat that was cooled to -20° C in advance. The mammary gland block was fixed by OCT embedding agent, and then 5 μM frozen section was made. Next, add 30 μL DAPI to the frozen section for 5 min, and then mount the slide. Obtain fluorescence images under a fluorescence microscope.

### Immunofluorescence measurement

Paraffin sections were immersed in xylene to deparaffinize, and then dehydrated with different concentrations of alcohol gradient. Next, add 50 μL of 0.1% Triton×100 solution to the slices for 1 h. Next, for antigen retrieval, the paraffin sections were immersed in a citrate solution and heated to 92° C, and the heating cycle was repeated twice. Then immerse the slices in the collodion solution for 15 min. Then they were blocked with PBST and 5% donkey serum for 1 h. Next, add the first antibody (1:100) and let it stand overnight at 4° C. On the second day, paraffin sections were added with secondary antibody (1:5000) for 1 h. Next, add 50 μL DAPI to the paraffin sections and mount the slides. Obtain experimental results under a fluorescence microscope. The first antibody was diluted with 5% BSA at the ratio of 1:100, occludin, clauding-3 were purchased from Abcam Co., Ltd. (Shanghai, China). Donkey anti-Rabit IgG (H+L) Highly Cross-Adsorbed Secondary Antibody, Alexa Fluor 594 was diluted with 5% donkey serum at the ratio of 1:1000, and it was purchased from Thermo Fisher Technology (Shanghai, China) Co., Ltd.

### Data and statistical analysis

ImageJ was used for image density analysis and image merge, Graphpad prism 8 was used for histogram statistics, Adobe Illustrator CS6 was used for data processing, and one-way Anova was used for data difference analysis. Values are presented as means ± SEM, three independent repeated experiments were performed; #p<0.01 vs. NT group; **p <0.01 vs. LPS group.

## RESULTS

### Effect of PE on the pathological damage of mammary gland in mice with mastitis

**Figure 1:**
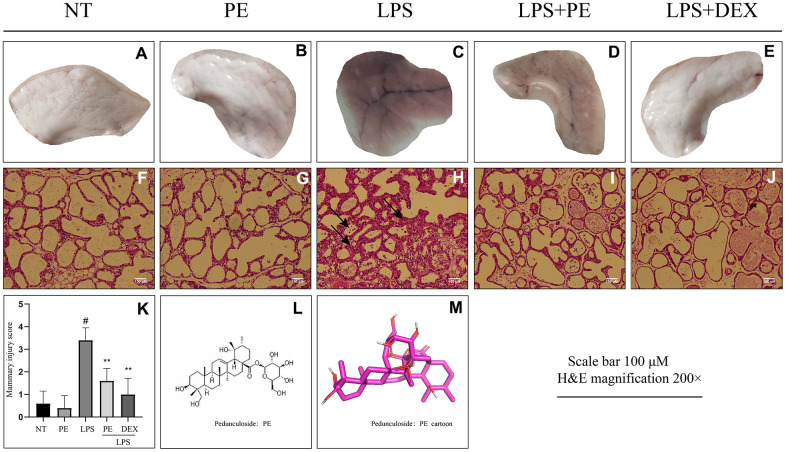
**Effect of Pedunculoside (PE) on the pathological damage of mammary gland in mice with mastitis.** PE (10 mg/kg) was given orally for 7 days before the establishment of mastitis model. The fourth pair of milk ducts in mice were injected with 50 μL of 0.2 μg/μL LPS for 24 h. The mice were killed by dislocation and fixed on the operating platform. The hair was disinfected and fixed by spraying 70% alcohol. The midline of abdomen was cut to expose the breast tissue. Finally, the mammary was photographed and collected. (**A**–**E**) Morphological photos of mouse mammary gland tissue; (**F**–**J**) H&E staining of mouse mammary gland paraffin section; (**K**) Pathological damage score of mice mammary gland tissue; (**L**) Structural formula of Pedunculoside; (**M**) 3D mode of Pedunculoside. The lesion was shown by the arrow in the figure. Scale bar 100 μM, H&E magnification 200×. Dexamethasone (DEX) was administered intramuscularly at a concentration of 5 mg/kg. Values are presented as means ± SEM, three independent repeated experiments were performed; #*p*<0.01 vs. No treatment group (NT) group; **p<0.01 vs. LPS group.

In order to clarify the effect of PE on mammary gland damage, we obtained the following experimental results through observation and H&E staining. From the visual point of view, compared with the no treatment group (NT) group, LPS significantly caused mammary gland redness and vascular congestion (Figure 1A, 1C). But the mammary injury was significantly relieved after PE pretreatment (Figure 1D). In addition, the effect of relieving mastitis of PE was similar as DEX (Figure 1D, 1E).

From the perspective of pathological damage, compared with the NT group, LPS significantly destroyed the structure of mammary tissue (Figure 1H). The pathological damage score of mouse mammary gland is shown in the figure (Figure 1K). The specific manifestations were the increase in the thickness of the acinar stroma, the acinar atrophy or even necrosis, and the infiltration of inflammatory cells in the acinar (Figure 1H, 1K). But the mammary injury was significantly relieved after PE pretreatment (Figure 1I, 1K), and the effect of relieving mastitis of PE was similar as DEX (Figure 1I–1K). In addition, the PE alone treatment group did not cause toxic side effects on the mammary both visually and under the microscope (Figure 1A, 1B, 1F, 1G, 1K).

### Effect of PE on inflammation level in mastitis model *in vivo* and *in vitro*

**Figure 2:**
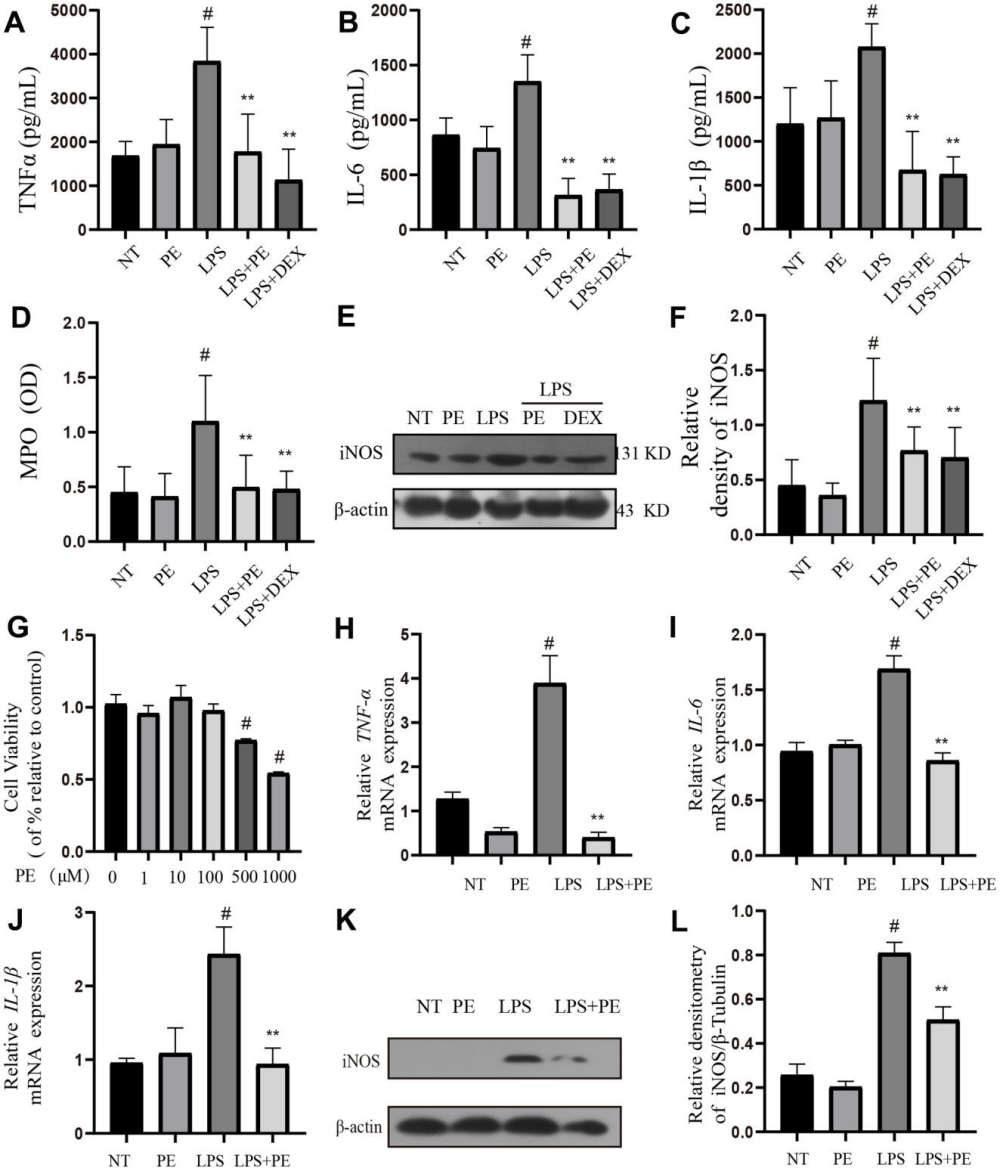
**Effect of PE on inflammation level in mastitis model *in vivo* and *in vitro*.** PE (10 mg/kg) was given orally for 7 days before the establishment of mastitis model. The fourth pair of milk ducts in mice were injected with 50 μL of 0.2 μg/μL LPS for 24 h. The mice were killed by dislocation and fixed on the operating platform. The hair was disinfected and fixed by spraying 70% alcohol. The midline of abdomen was cut to expose the breast tissue. Finally, the mammary was collected. mMECs was pretreatmented with 10 μM PE for 1 h, and then LPS stimulated the cells for 12 h. The inflammatory mediator’s levels of mice mammary gland and mouse mammary epithelial cells (mMECs) were measured by ELISA, MPO activity assay, western blot or qRT-PCR. (**A**–**C**) TNF-α, IL-6 and IL-1β protein content in mammary gland; (**D**) MPO content in mammary gland. (**E**, **F**) Western blot assay of iNOS protein in mammary tissue and in mMECs. (**G**) The activity of mMECs. (**H**–**J**) *TNF-α, IL-6, IL-1β* gene expression level in mMECs. (**K**, **L**) Western blot assay of iNOS protein in mMECs. Values are presented as means ± SEM, three independent repeated experiments were performed. #*p*<0.01 vs. NT group; **p < 0.01 vs. LPS group.

In order to confirm the effect of PE on inflammation, we further studied its effect on inflammation-related indicators *in vivo* and *in vitro*. First, *in vivo* experiments we found that compared with the NT group, LPS significantly increased inflammatory mediators IL-1β, TNF-α, IL-6, iNOS protein levels and MPO activity, but PE pretreatment abolished these effects of LPS (Figure 2A–2F), and the effect of PE is similar to that of DEX (Figure 2A–2F).

Then, *in vitro*, we used LPS to stimulate mMECs cells to establish a cellular inflammatory model, and the inflammatory indexes were detected *in vivo*. The experimental results showed that compared with the NT group, LPS significantly increased inflammatory mediators *IL-1β, TNF-α, IL-6* gene and iNOS protein levels, but PE pretreatment abolished these effects of LPS (Figure 2H–2L). In addition, PE within 100 μM has no toxic effect on mammary epithelial cells (Figure 2G), 10 μM PE was used for pre-protection, in the cell experiments, and the PE group alone did not cause an increase of cytokines *in vivo* and *in vitro*. These results indicated that the mastitis model was successfully constructed, and PE alleviated mastitis by inhibiting the increase of inflammatory mediators.

### Effect of PE on AKT/NF-κB signal pathway in mastitis models *in vivo* and *in vitro*

**Figure 3:**
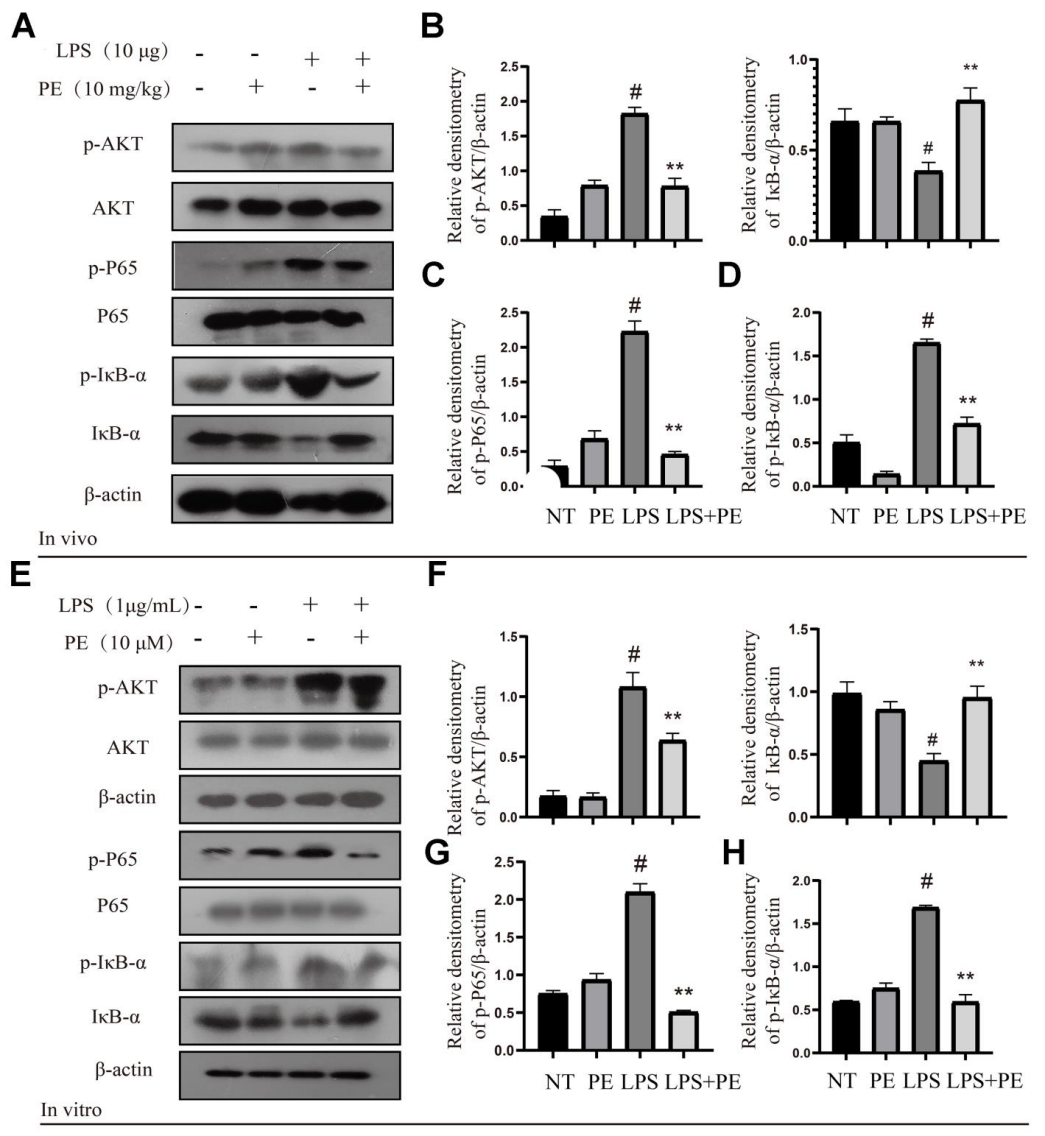
**Effect of PE on AKT/NF-κB signal pathway in mastitis models *in vivo* and *in vitro*.** PE was given orally for 7 days. The fourth pair of milk ducts in mice were injected with LPS for 24 h. The mice were killed by dislocation and fixed on the operating platform after LPS injection 24 h. The midline of abdomen was cut to collect mammary gland. PE was added to the cell culture medium at a concentration of 10 μM. After 1 h, LPS was added to the culture medium at a concentration of 1 μg/mL. The co-stimulation time was 12 h. (NP40) was added to mice mammary gland and mMECs, and then Western blot samples were prepared to obtain protein bands. (**A**–**D**) Western blot assay of p-AKT, AKT, p-P65, P65, p-IκB-α and IκB-α in mammary gland. (**E**–**H**) Western blot assay of p-AKT, AKT, p-P65, P65, p-IκB-α, IκB-α in mMECs. Each immunoreactive band was digitized and expressed as a ratio of the β-actin level. Values are presented as means ± SEM, three independent repeated experiments were performed. #*p*<0.01 vs. NT group; ^**^p < 0.01 vs. LPS group.

In order to explore the potential mechanism of PE alleviating mastitis, we tested the pro-inflammatory classic signaling pathway AKT/NF-κB *in vivo* and *in vitro*. The results showed that compared with the NT group, LPS significantly activated the phosphorylation level of AKT protein *in vivo* and *in vitro*, and PE pretreatment effectively alleviated the phosphorylation level of AKT protein (Figure 3A, 3B, 3E, 3F). In addition, we further explored the pro-inflammatory signal NF-κB located downstream of AKT. Our results showed that LPS significantly activated the phosphorylation level of P65 and IκB protein *in vivo* and *in vitro*, and promoted the ubiquitination and degradation of IκB protein, but PE pretreatment abolished this effect of LPS (Figure 3A, 3C–3E, 3G, 3H; Figure 8). These findings indicated that PE inhibits the activation of NF-κB signal by inhibiting the phosphorylation level of AKT protein, effectively alleviating the exacerbation of the inflammatory response in mammary tissue.

### Effect of PE on MAPK signal pathway in mastitis models *in vivo* and *in vitro*

**Figure 4:**
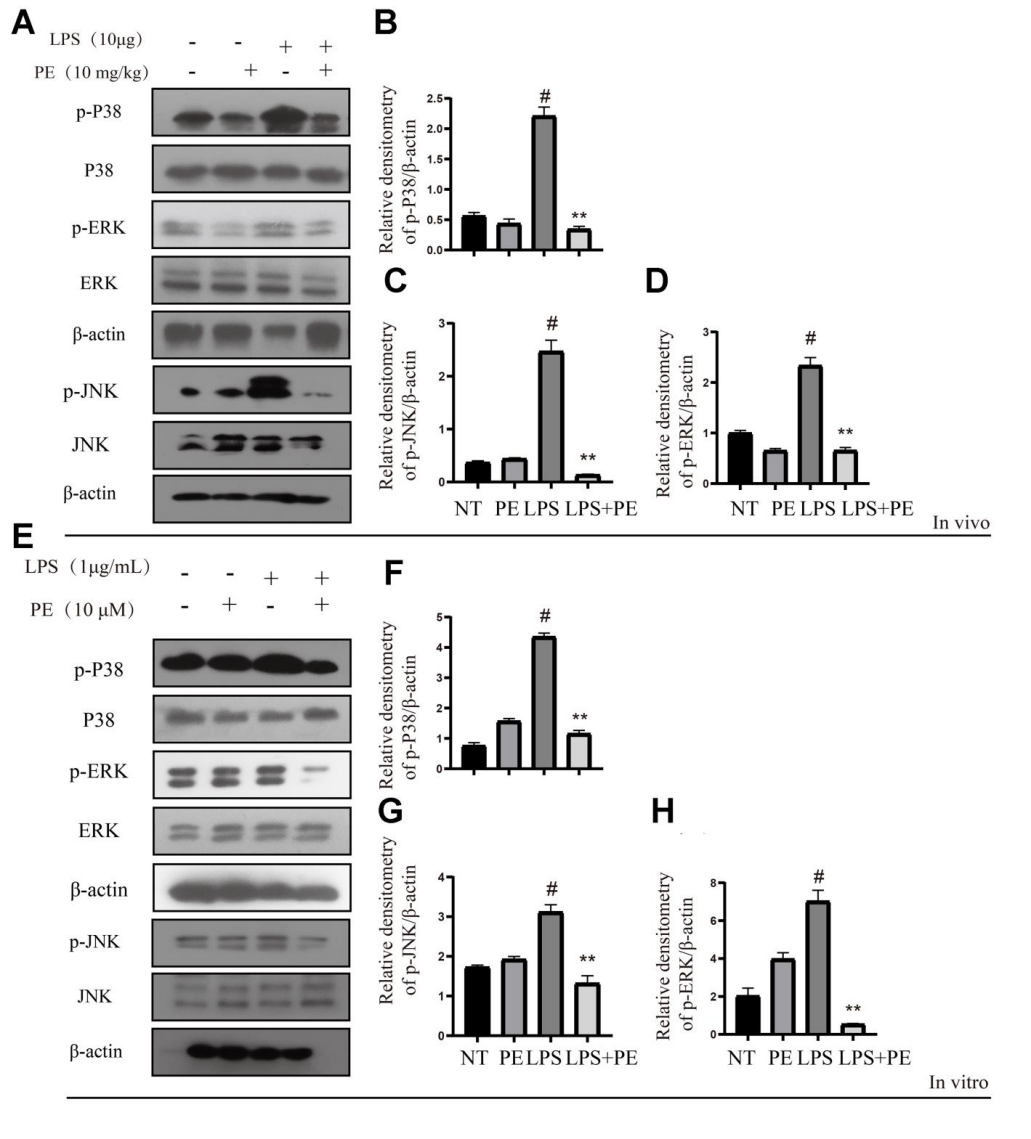
**Effect of PE on MAPK signal pathway in mastitis models *in vivo* and *in vitro*.** PE was given orally for 7 days. The fourth pair of milk ducts in mice were injected with LPS for 24 h. The mice were killed by dislocation and fixed on the operating platform after LPS injection 24 h. The midline of abdomen was cut to collect mammary gland. PE was added to the cell culture medium at a concentration of 10 μM. After 1 h, LPS was added to the culture medium at a concentration of 1 μg/mL. The co-stimulation time was 12 h. (NP40) was added to mice mammary gland and mMECs, and then Western blot samples were prepared to obtain protein bands. (**A**–**D**) Western blot assay of p-P38, P38, p-JNK, JNK, p-ERK and ERK in mammary gland. (**E**–**H**) Western blot assay of p-P38, P38, p-JNK, JNK, p-ERK and ERK in mMECs. Each immunoreactive band was digitized and expressed as a ratio of the β-actin level. Values are presented as means ± SEM, three independent repeated experiments were performed. #*p*<0.01 vs. NT group; ^**^p < 0.01 vs. LPS group.

MAPK is another important pro-inflammatory signaling pathway, which is located downstream of AKT signal [22]. In order to further explore the potential mechanism of PE alleviating mastitis, we tested the phosphorylation level of MAPK signal. Our research found that LPS significantly activated the phosphorylation levels of P38, ERK1/2, and JNK proteins *in vivo* and *in vitro*, but PE pretreatment abolished this effect of LPS (Figure 4A–4H; Figure 8). The results of this study indicated that PE inhibited the inflammatory response by inhibiting the phosphorylation level of MAPK signal, effectively alleviating the exacerbation of the inflammatory response in mammary tissue.

### Molecular dynamics simulation of the binding site of PE and AKT protein

**Figure 5:**
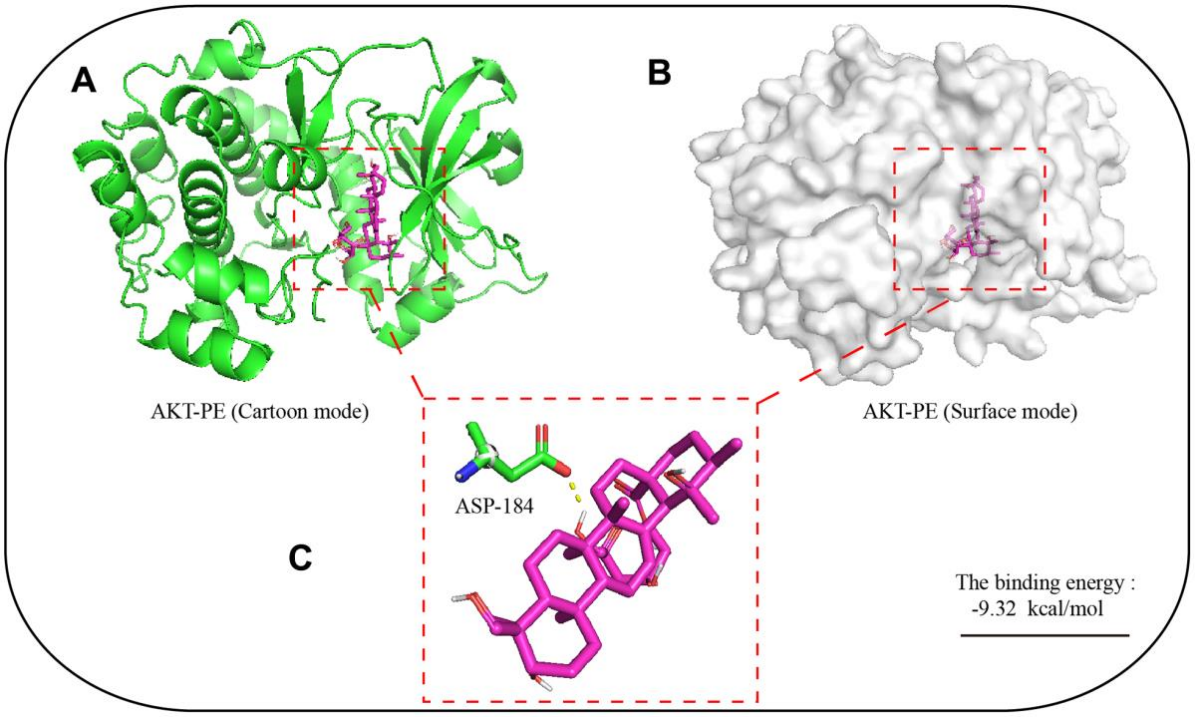
**Molecular dynamics simulation of the binding site of PE and AKT protein.** The 3D structure of PE was obtained from PubChem. The initial X-ray crystal structure of AKT was obtained from the Protein Data Bank. (**A**) 3D structure of AKT with PE (Cartoon mode); (**B**) 3D structure of AKT with PE (Surface mode); (**C**) PE and AKT proteins are predicted to bind to the amino acid residue aspartic acid 184 (ASP-184); The autodock4 software was used for molecular docking simulation, and 2.5x10^6^ operations were executed, used to find potential docking sites.

In order to explore the potential binding mode of PE and AKT protein, this experiment carried out molecular dynamics simulation based on molecular docking technology. The binding mode of PE and AKT was shown in Figure 5, we clearly see that there was a hydrogen bond interaction between PE and AKT protein, which hydrogen bonding between ASP-184 residue of AKT protein and PE. We calculated that the total binding energy of PE and AKT was -9.32 kal/moL, and the sum energy was less than 0 kal/moL with statistical significance.

### The effect of PE on the integrity of blood-milk barrier in mice with mastitis

In order to further explore the effect of PE on mastitis, we detected the effect of PE on blood-milk barrier integrity. Fresh mice mammary gland was immersed in a 2 mg/mL FITC solution, quickly frozen in liquid nitrogen, and then frozen sections were prepared and obtained to evaluate the damage of the mice blood-milk barrier. Green fluorescence represents albumin fluorescein isothiocyanate conjugate protein province (FITC); blue represents nucleus. The arrows represent the leaking.

**Figure 6:**
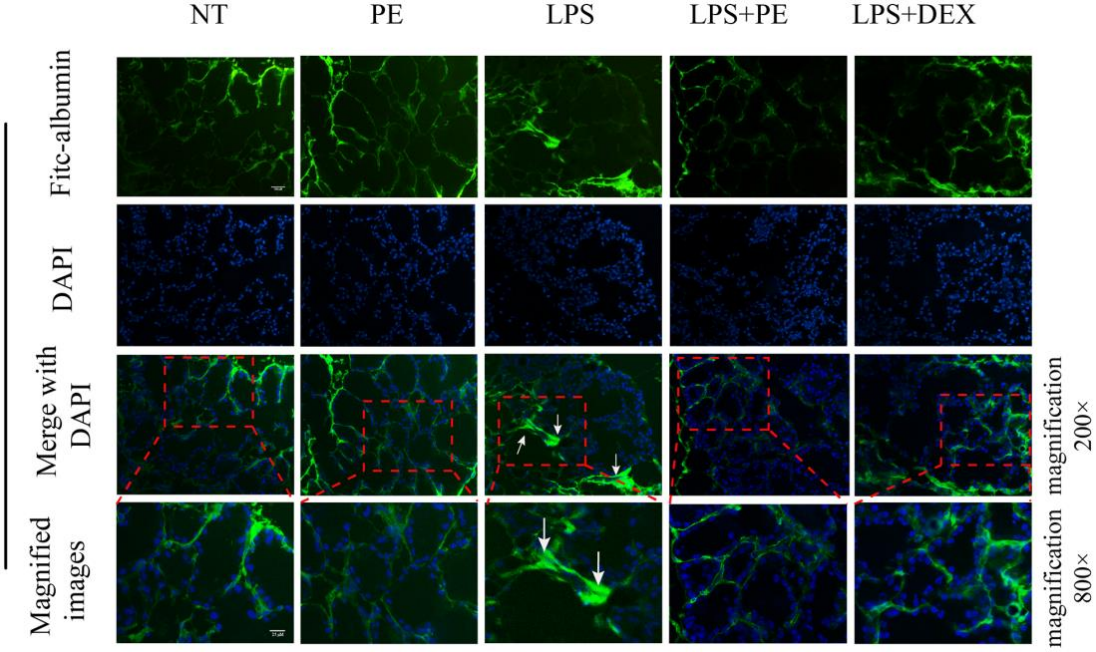
**Effect of PE on the integrity of blood-milk barrier in mice with mastitis *in vivo*.** Fresh mice mammary gland was immersed in a 2 mg/mL FITC solution, quickly frozen in liquid nitrogen, and then frozen sections were prepared and obtained to evaluate the damage of the mice blood-milk barrier. Green fluorescence represents albumin fluorescein isothiocyanate conjugate protein province (FITC); blue represents nucleus. The arrows represent the leaking FITC in the damaged acinus and the shrinking acinar. The scale bar was 25 and 100 μM, respectively.

Studies have shown that inflammation destroy the blood-milk barrier and repairing the blood-milk barrier is beneficial to alleviate the symptoms of mastitis. Our research results showed that compared with the NT group, the blood-milk barrier of the LPS group was significantly damaged. The FITC-white permeation from the inter-acinular matrix was detected in the acinar cavity. The damage of protein and blood-milk barrier was shown by the arrow in the figure (Figure 6A). This result shows that PE significantly reverses the damage of LPS to the blood-milk barrier, thereby alleviating the severity of mastitis.

### The effect of PE on tight junction proteins occludin and claudin-3

**Figure 7:**
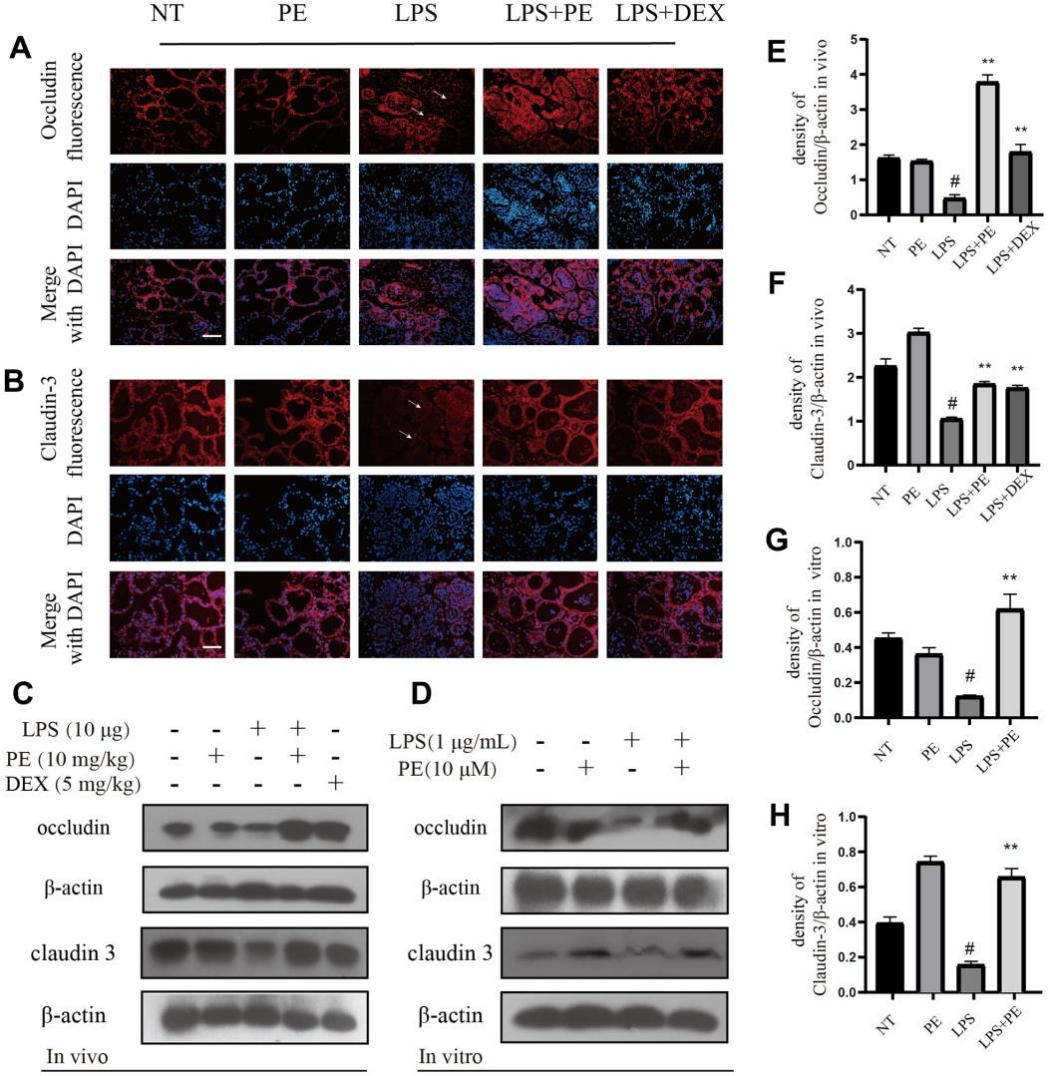
**The effect of PE on tight junction proteins occludin and claudin-3.** PE was given orally for 7 days. The fourth pair of milk ducts in mice were injected with LPS for 24 h. The mice were killed by dislocation and fixed on the operating platform after LPS injection 24 h. The midline of abdomen was cut to collect mammary gland. PE was added to the cell culture medium at a concentration of 10 μM. After 1 h, LPS was added to the culture medium at a concentration of 1 μg/mL. The co-stimulation time was 12 h. (NP40) was added to mice mammary gland and mMECs, and then Western blot samples were prepared to obtain protein bands., Immunofluorescence and Western blot methods were used to evaluate the expression changes of tight junction proteins occludin and claudin-3 in mammary gland and mMECs. Immunofluorescence of occludin (**A**) and claudin-3 (**B**) in frozen sections of mammary gland. Scale bar 100 μM. (**C**, **E**, **F**) Western blot assay of occludin and claudin-3 in mammary gland; (**D**, **G**, **H**) Western blot assay of occludin and claudin-3 in mMECs. Each immunoreactive band was digitized and expressed as a ratio of the β-actin level. Values are presented as means ± SEM, three independent repeated experiments were performed; #*p*<0.01 vs. NT group; ^**^p < 0.01 vs. LPS group.

**Figure 8:**
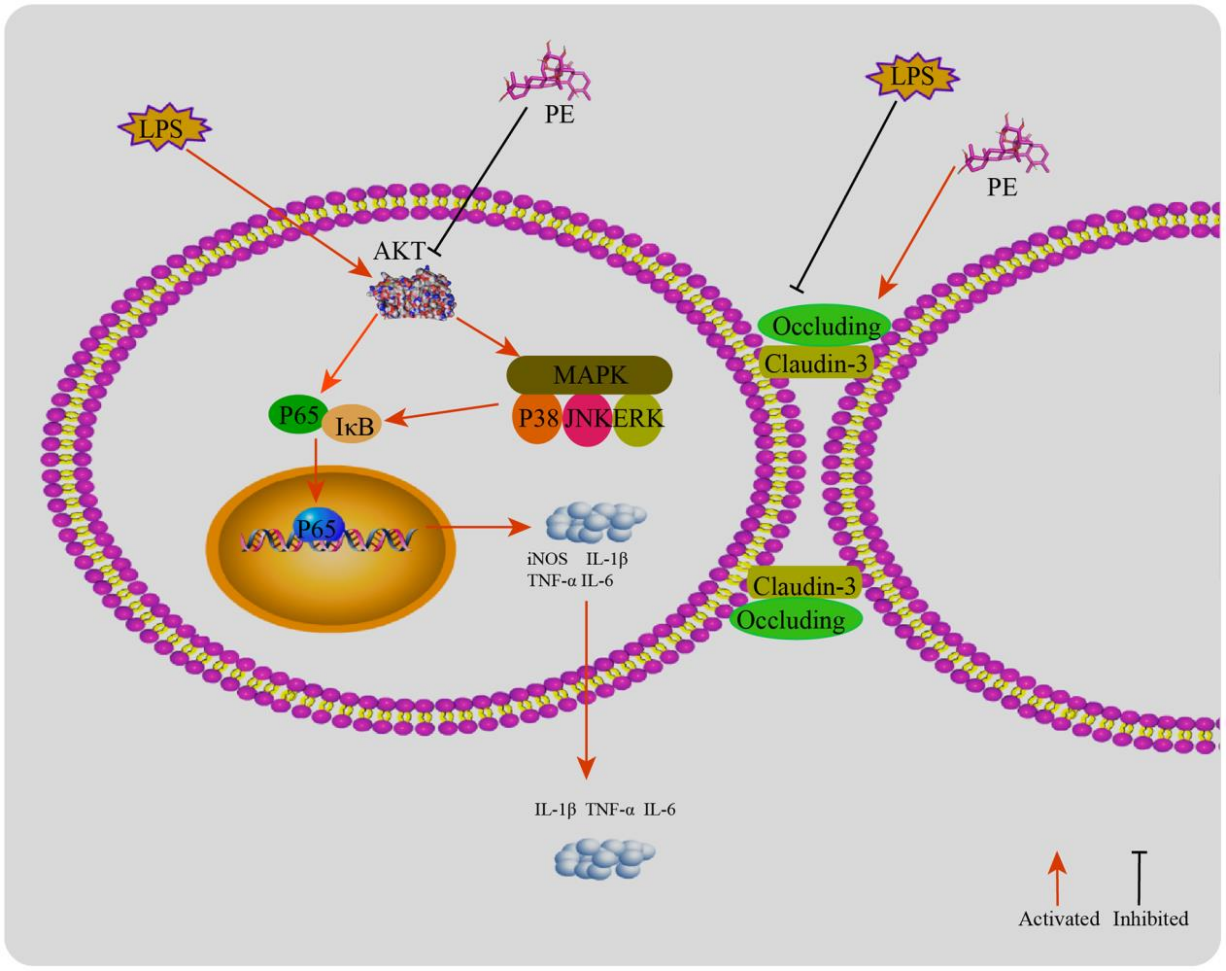
**The mechanism of PE alleviating mastitis in LPS-induced mastitis.** LPS activates the inflammatory response and destroys the blood-milk barrier. PE inhibits the inflammatory response by inhibiting the AKT/NF-κB and MAPK signaling pathways. ASP-184 may be a potential binding site for PE to inhibit AKT protein; In addition, PE promotes the tight junction proteins occludin and claudin-3 expression, repair the blood and mammary barrier, and relieve mastitis.

In order to further explore the potential mechanism of PE repairing the blood-milk barrier in the course of mastitis, we tested the effect of PE on tight junction proteins. Our research results showed that compared with the NT group, the occludin protein of LPS group was significantly damaged, and the distribution of the occludin protein in the mammary acinar was scattered and irregular as shown by the arrow in the figure (Figure 7A; Figure 8). But the PE pretreatment significantly promoted the tissue integrity of occludin and promote its protein expression. Similarly, PE effectively alleviate the decrease in claudin-3 protein expression and the disordered tissue distribution caused by LPS (Figure 7A; Figure 8). In addition, our western blot results further confirmed that PE significantly reversed the decreasing of occludin and claudin-3 protein levels induced by LPS (Figure 7C–7H; Figure 8) *in vivo* and *in vitro*. These results indicated that PE repair the blood-milk barrier by promoting the expression of tight junction proteins occludin and claudin-3.

## DISCUSSION

Mastitis is a complex pathological process strong inflammatory reaction is one of the main symptoms of the disease [1, 8]. In recent years, blood-milk barrier has become another important research direction of mastitis [23]. In addition, food additives have a good effect in preventing mastitis, which is a new research direction. Therefore, some food supplement that can inhibit the inflammatory response and repair the blood-milk barrier may be a potential drug to alleviate mastitis. The results of this experiment shown that PE significantly inhibited the inflammatory response in the process of mastitis and promoted the repair of the blood-milk barrier, and further explore its underlying mechanism.

Inflammatory mediators are important effector factors in the process of inflammatory reaction, such as IL-6, IL-1β, TNF-α, iNOS, MPO and so on. The rapid release of a large number of inflammatory mediators will break the balance beyond the mammary immune system, recruit a large number of neutrophils, lead to the damage of mammary cell function and mammary gland damage [24, 25]. On the other hand, inflammatory mediators stimulate mammary duct swelling, resulting in milk stasis, and then aggravate the inflammatory response of the mammary gland [26]. Therefore, effective inhibition of inflammatory cytokine levels is beneficial to alleviate the symptoms of mastitis. Studies have shown that butyric acid can effectively alleviate LPS-induced mastitis in mice by inhibiting the increase in cytokine levels [9]. Our results shown that PE effectively alleviate mammary inflammation by inhibiting the expression and secretion of IL-6, IL-1β, TNF-α *in vivo* and *in vitro*, and inhibiting the synthesis of iNOS and MPO.

AKT, also known as protein kinase B, is closely related to the pathological process of cells, such as inflammation and apoptosis [27]. The activation of AKT can transmit inflammatory signal down, promote the release of inflammatory mediators and intensify the inflammatory response [28]. Studies have shown that inhibiting the activation of AKT protein effectively alleviate mastitis disease, so AKT is used as a potential therapeutic target for the treatment of mastitis [23]. Our research shown that PE effectively inhibited the activation of AKT protein, and its potential binding site is located at ASP-184 amino acid residues. NF-κB is an important inflammatory signal pathway, located downstream of the AKT signal. The translocation of P65 protein into the nucleus can directly promote the transcription and translation of inflammatory cytokines IL-6, IL-1β, and TNF-α, and aggravate the inflammatory response [23, 29]. AKT/NF-κB is often used as an important anti-inflammatory signal axis. Studies have shown that inhibiting the activation of AKT/NF-κB signal can effectively inhibit the occurrence of mammary inflammatory diseases [3, 23]. Our results shown that PE effectively inhibited the activation of AKT/NF-κB signal and alleviated LPS induced mastitis.

MAPK is another important pro-inflammatory signaling pathway, which is located downstream of AKT signal (Populo et al., 2012). LPS needs this signal to induce the production of pro-inflammatory enzymes and cytokines in mammary epithelial cells [23, 30]. Therefore, in order to further explore the potential mechanism of PE to prevent mastitis, we explored the effect of PE on the phosphorylation of MAPK-related proteins. As expected, PE effectively inhibited the phosphorylation levels of P38, ERK1/2, and JNK proteins. Research results indicate that activation of MAPK will further promote the activation of pro-inflammatory transcription factor NF-κB, and aggravate the severity of the inflammatory response [5]. In addition, some studies have shown that inhibiting the phosphorylation level of MAPK protein can effectively alleviate the symptoms of mastitis [8, 23]. Therefore, inhibiting the activation of MAPK signaling pathway is another important potential therapeutic target for PE to alleviate mastitis.

Blood-milk barrier is an important biological barrier between blood and milk, which is mainly the tight junction structure between epithelial cells composed of tight junction proteins [23, 31]. The integrity of the blood-milk barrier and the dynamic balance of tissue permeability are key factors for maintaining mammary health [31, 32]. Studies have shown that good blood-milk barrier can effectively resist the invasion of pathogens and prevent the loss of nutrients in milk [32]. In addition, studies have shown that the integrity of the blood-milk barrier is damaged in the course of mastitis [9, 23]. It is interesting to note that repairing the blood-milk barrier during the mastitis process is beneficial to promote the cure of mastitis. Our research results also show that PE effectively promotes the repair of the blood-milk barrier, and its specific mechanism is to promote the expression of tight junction proteins occludin and claudin-3. Therefore, we found that another important mechanism of PE to prevent mastitis is the protection of the blood-milk barrier.

In conclusion, PE significantly alleviate the symptoms of mastitis, and its potential mechanism is that the inhibition of the activation of AKT/NF-κB and MAPK signaling pathways, therefore controls the inflammatory response; In addition, PE promotes the expression of tight junction protein, repairs the blood-milk barrier, and then alleviates mastitis.
